# Effect of Functional Groups in Lipid Molecules on the Stability of Nanostructured Lipid Carriers: Experimental and Computational Investigations

**DOI:** 10.1021/acsomega.4c00685

**Published:** 2024-02-21

**Authors:** Warangkana Pornputtapitak, Yenruedee Thiangjit, Yuthana Tantirungrotechai

**Affiliations:** † Department of Chemical Engineering, Faculty of Engineering, Mahidol University, Nakhon Pathom 73170, Thailand; ‡ Thammasat University Research Unit in Innovation of Molecular Hybrid for Biomedical Application and Division of Chemistry, Faculty of Science and Technology, Thammasat University, Pathum Thani 12120, Thailand

## Abstract

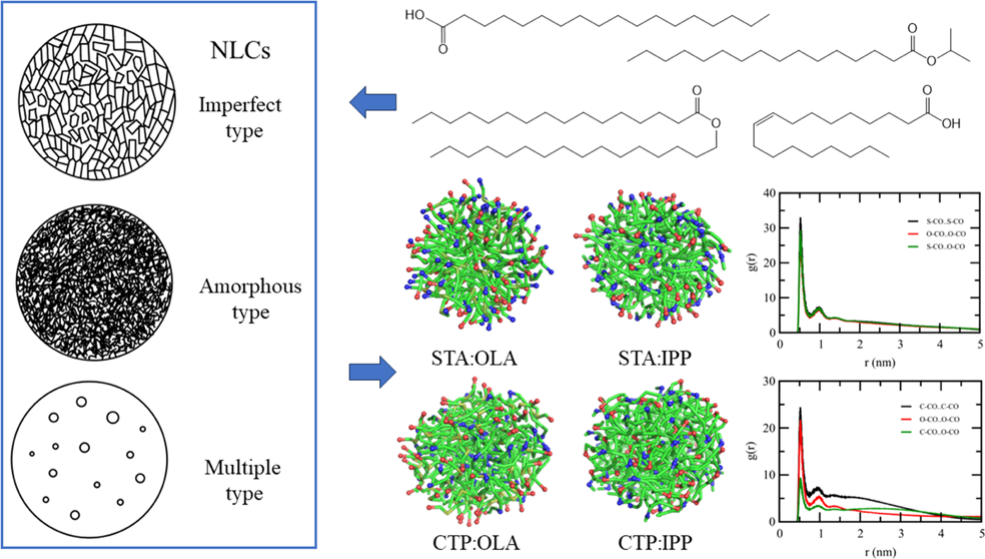

Lipid nanoparticles have been used as drug carriers for decades. Many lipid types have been screened for both solid lipid nanoparticles and nanostructured lipid carriers (NLCs). Specifically, for NLCs that are composed of lipids in the solid form mixed with lipids in the liquid form, compatibility of lipid combination and phase behavior play a significant role in the NLC quality. In this study, stearic acid (STA) and cetyl palmitate (CTP) were used as solid lipids, and oleic acid (OLA), isopropyl palmitate (IPP), and caprylic/capric triglycerides were used as liquid lipids. NLCs were prepared at solid:liquid ratios of 50:50, 70:30, and 90:10, respectively. The characteristics and stability of the prepared NLCs were investigated. Laboratory results showed that the solid lipid had a greater influence on the particle size than the liquid lipid. Meanwhile, cetyl palmitate, an ester compound, provides higher NLC stability compared to stearic acid, a carboxylic acid compound. A MARTINI-based coarse-grained molecular dynamics simulation was used to simulate the lipid droplet in water. The distribution of lipid molecules in the droplet was characterized by the polar group density distribution. Different spatial arrangements of the lipid headgroup and lipid molecules, when CTP or STA was used as solid lipids, might contribute to the different stabilities of prepared NLCs. The understanding of mixed lipid systems via simulations will be a significant tool for screening the type of lipids for drug carriers and other pharmaceutical applications.

## Introduction

A lipid nanoparticle was developed as an alternative drug carrier for administration routes including oral,^1^ topical,^2^ and pulmonary.^3^ The developed nanoparticle was biocompatible and had low toxicity. The first generation comprised solid lipid nanoparticles (SLN). This lipid is in solid form at room temperature and is the main component of SLN. It is the only solid lipid that will be recrystallized during storage, resulting in drug expulsion from the SLN matrices. In general, the drug or active compound is held between the fatty acid chains or lipid layers in amorphous clusters. To increase the amount encapsulated in the lipid matrix, the number of amorphous clusters can be increased. This is a promising concept for the development of lipid nanoparticles as a carrier. The second generation comprised nanostructured lipid carriers (NLCs). These were developed to overcome the limitations of SLN.^4^ NLCs combine solid and liquid lipids. The encapsulation efficiency increases when NLCs are used as the carriers because the crystallinity of the lipid matrix is decreased when a liquid lipid is added. NLCs, therefore, help to prevent the release of the drug during storage.^5,6^ However, as NLCs are composed of both solid lipids and liquid lipids, the composition has an influence on its characteristics. From the literature, the structure of the NLC matrix depends on the types of lipids used. NLCs can be categorized into three groups. The first group is an imperfect crystal that is formed when the solid and liquid lipids have different side chain lengths. The imperfect crystal is also formed when mixtures of mono-, di-, and triglyceride are used to prepare the NLCs.^7^ The second NLC group is in the amorphous form. It forms when types of lipids such as hydroxyl stearate or isopropyl myristate are used. The last group, called a multiple model, occurs when long-chain lipids are used as both solid and liquid lipids, or oleic acid is used as a liquid lipid.^8^

A previous study reported that the lipid type had an influence on the encapsulation efficiency of SLN but had no effect on the chemical stability.^9^ Different types of solid and liquid lipids can be matched. These include topotecan-loaded lipid nanoparticles composed of oleic acid and stearic acid.^10^ Rehman et al. prepared the binary fatty acid mixture-based solid lipid nanoparticles for the delivery of diacerein, an osteoarthritis drug.^11^ The binary lipid mixtures were prepared using different ratios of solid (stearic or lauric acid) and liquid (oleic acid) fatty acid. The SLN particle size depends on the preparation technique, with the solvent emulsification–evaporation technique yielding the smallest size around 8 nm. Several studies have used cetyl palmitate as the solid lipid and caprylic/capric triglycerides as the liquid lipid.^12,13^ In the current study, since the lipid type plays an important role in the quality of the NLCs, the effect of the lipid type on the quality, and especially the size and stability of the prepared NLCs, was studied.

In addition to NLC preparation, a coarse-grained (CG) molecular dynamics simulation was performed to understand the mixing compatibility and the local structure of solid and liquid lipids at the molecular level. The MARTINI coarse-grained force field was used to represent the fatty acid molecules to speed up the calculation. The MARTINI force field is known to provide the correct behavior of cellular membranes and other biomolecular structures while reducing the degrees of freedom and covering a longer simulation period.^14−16^ Janke et al. investigated the phase behavior of oleic/oleate systems using the MARTINI force field.^17^ The oil phase is formed when the fatty acids are in a protonated state. The structure forms vesicles and then wormlike micelles when the ratio of deprotonated/protonated fatty acids is increased. Bennett et al. investigated the oleic acid aggregates in water.^18^ They conducted a constant pH simulation with 20–30 oleic acids in water using the coarse-grained MARTINI force field. The oleic acid molecules could ionize according to the pH value. The aggregation of oleic acid is spontaneous. The micelle size is dependent on the pH or the degree of deprotonated oleic acid in the system. Hossain et al. studied the aggregation behavior of four different types of medium-chain fatty acids using coarse-grained molecular dynamics simulation.^19^ The authors varied the number of free fatty acid molecules to determine the critical micelle concentration. The aggregate sizes and morphologies of the coarse-grained particles are consistent with the experimental observation. Larsson et al. performed a coarse-grained molecular dynamics simulation to understand the internal arrangement of lipid-based formulations dispersed in water.^20^ The local ordering in the lipid mixture and the phase change upon water dispersion can be depicted by coarse-grained simulation.

Lipids used for NLC preparation can be categorized into four main groups: those that have a functional alcohol group, carboxylic group, or ester group in the molecule, and those in the form of triglycerides. From preliminary studies, lipids with ester groups formed smaller and more stable particles than did lipids with alcohol or carboxylic groups. In this study, solid and liquid lipids containing an ester group and a carboxylic group were used for NLC preparation. The caprylic/capric triglycerides that are normally used for preparing NLCs were also studied. The coarse-grained molecular dynamics simulation based on the MARTINI force field was carried out to understand how lipids with different functional groups affect the characteristics of NLCs.

## Results and Discussion

### Effect of Lipid Type

**Figure 1:**
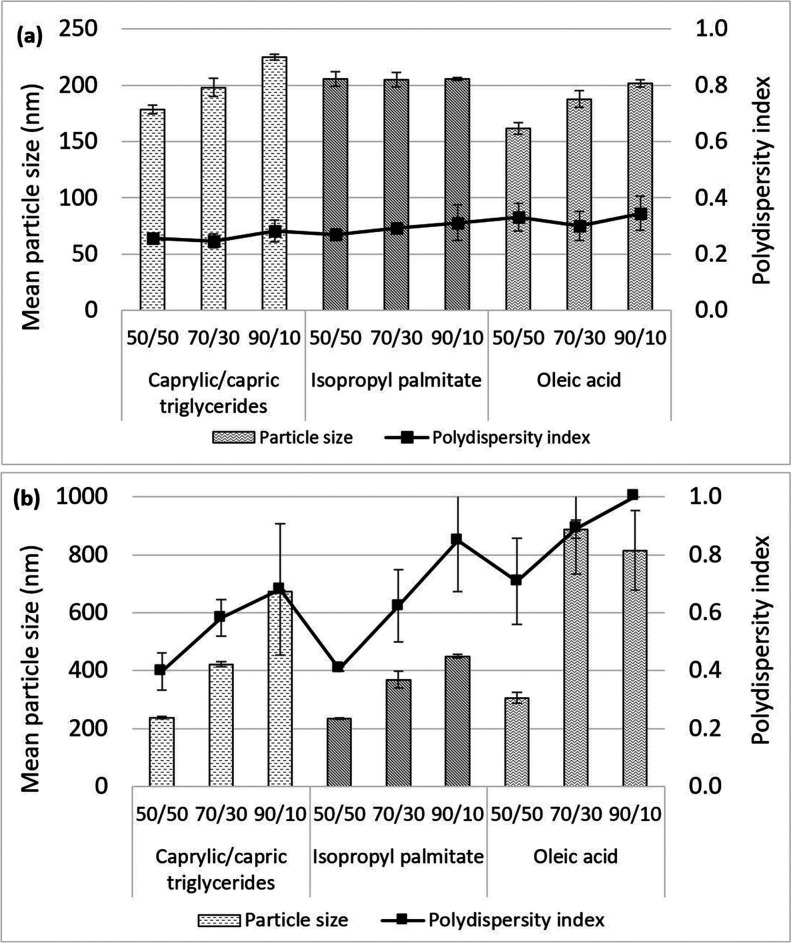
Mean particle size and polydispersity index of NLCs prepared using different lipid combinations. (a) Cetyl palmitate as a solid lipid and (b) stearic acid as a solid lipid.

NLC formulations were prepared by matching the types of solid and liquid lipids. Cetyl palmitate (an ester compound) and stearic acid (an acid compound) were used as solid lipids, while isopropyl palmitate (an ester compound), oleic acid (an acid compound), and caprylic/capric triglycerides were used as liquid lipids. The particle size and polydispersity (PI) of the prepared NLCs are shown in Figure 1.

NLCs formulated using stearic acid showed significantly larger particle sizes (*p* < 0.05) than NLCs from cetyl palmitate in all formulations (Figure 1a,b). The use of cetyl palmitate as the solid lipid generated NLCs in the range of 161.67 ± 4.92 to 225.13 ± 2.76 nm. The mean particle size did not change significantly as the liquid lipid types varied but increased slightly when the amount of solid lipid in the NLCs was increased. However, this effect depended on the type of liquid lipid used. When caprylic/capric triglycerides and oleic acid were used, the mean particle size increased gradually as the concentration of cetyl palmitate was increased from 50 to 70 and 90% (Figure 1a). The particle size did not change as the solid/liquid lipid ratio was varied when isopropyl palmitate was used as the liquid lipid. The polydispersity index (PI) was in the range of 0.25 ± 0.02 to 0.35 ± 0.06, indicating the optimum particle dispersion.^21^ The PI has a range from 0 to 1, and a lower value represents better particle dispersion. The zeta potentials of the NLC formulations were in the range of −25.33 ± 0.38 to −34.37 ± 0.64 mV, which would prevent particle aggregation during storage. Particles with a zeta-potential lower than −15 mV should be stable, whereas gelation phenomena may occur at higher values.^5^ Moreover, Span40 and Tween80 are nonionic surfactants that can prevent agglomeration of NLCs via steric stabilization.^21^

NLCs formed using stearic acid rather than cetyl palmitate showed an influence from both the solid and liquid lipids (Figure 1b). The particle size was significantly increased for all liquid lipid types as the ratio of the solid:liquid lipid was increased. The PI changed from 0.40 ± 0.06 to 1, suggesting poor particle dispersion, especially at a solid-to-liquid lipid ratio of 90:10 when oleic acid was used as the liquid lipid.

Among the three liquid lipid types investigated, isopropyl palmitate was the most promising one. Increasing the proportion of stearic acid slightly affected the particle size, whereas this did not change as the concentration of cetyl palmitate was increased. The use of oleic acid and caprylic/capric triglycerides had no effect on the particle size when cetyl palmitate was used as the solid lipid. Oleic acid showed the largest particle size and the highest PI when stearic acid was used as the solid lipid.

### Stability Study

**Figure 2:**
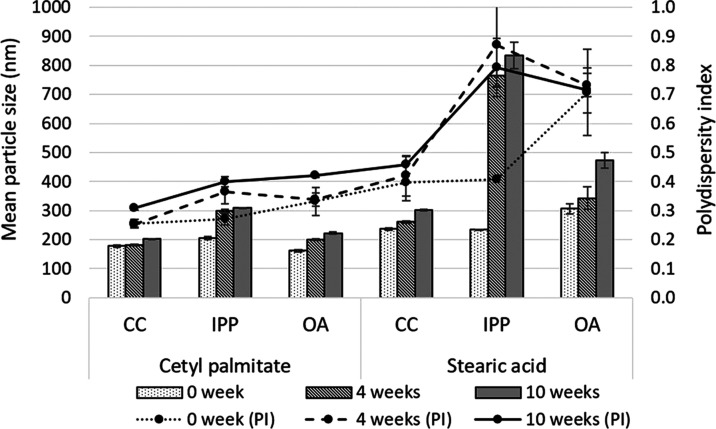
Mean particle size and polydispersity index of NLCs prepared using different lipid combinations at a solid:liquid lipid ratio of 50:50.

The NLC formulations were stored at 4 °C for 3 months. The mean particle size was measured at the fourth and tenth weeks of storage. The results showed that the mean particle size and PI slightly increased as the storage time increased. At a solid-to-liquid lipid ratio of 90:10, the particle size and PI increased in all formulations. At a ratio of 50:50, the most significant change in particle size was found when isopropyl palmitate was used as the liquid lipid and stearic acid as the solid lipid (Figure 2).

When cetyl palmitate was used as the solid lipid, the particles increased in size by approximately 100 nm by 10 weeks. When stearic acid was used as the solid lipid, its concentration in the formulation had a greater effect than the storage time, except when isopropyl palmitate was used as the liquid lipid. In this case, a significant increase of approximately 600–1000 nm was found over 10 weeks in all formulations at all solid-to-liquid lipid ratios. When cetyl palmitate was used as the solid lipid, the greatest stability was found for the formulation that mixed caprylic/capric triglycerides at solid-to-liquid lipid ratios of 50:50 and oleic acid at 50:50 and 70:30. When stearic acid was used as the solid lipid, the most stable formulations mixed caprylic/capric triglycerides and oleic acid in a ratio of 50:50.

### Differential Scanning Calorimetry (DSC) Analysis

**Figure 3:**
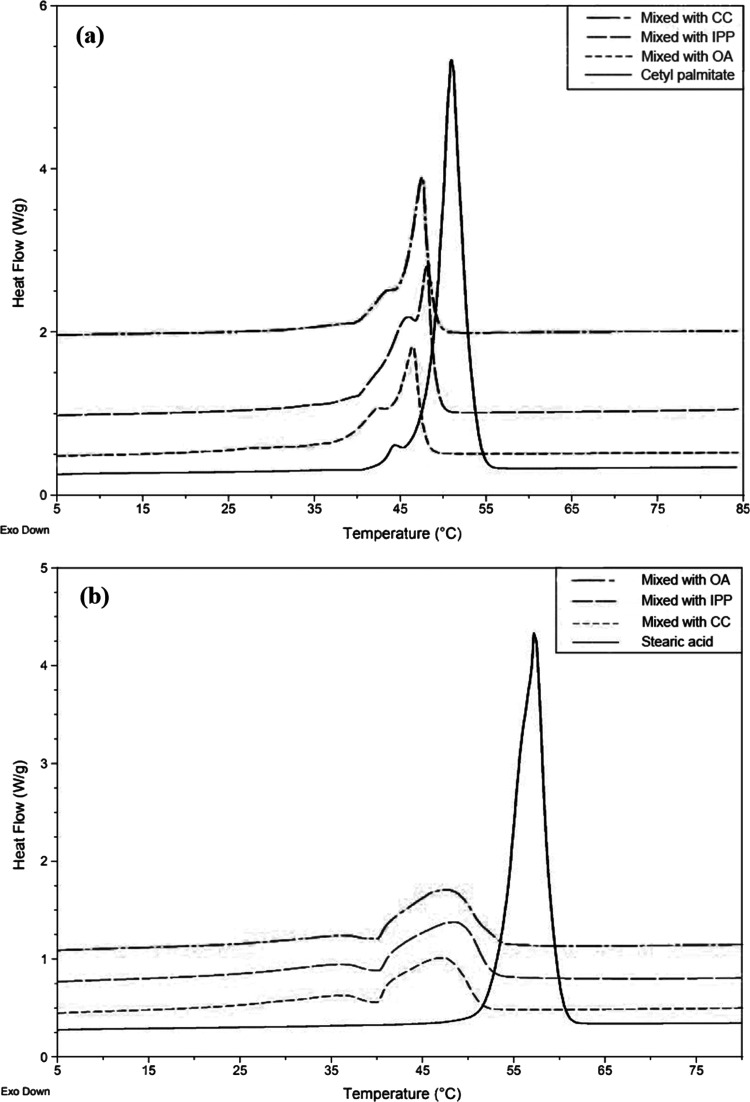
DSC thermogram of NLC formulations composed of (a) cetyl palmitate and (b) stearic acid mixed with caprylic/capric triglycerides (CC), isopropyl palmitate (IPP), and oleic acid (OA) at a solid-to-liquid lipid ratio of 70:10.

From the DSC thermogram, the melting peaks of the NLCs were at lower temperatures with smaller changes in heat flow than those of bulk cetyl palmitate or stearic acid, indicating lower crystallinity (Table 1). When cetyl palmitate was mixed with liquid lipids, the crystallinity decreased, although sharp peaks were still observed in all three mixtures. Generally, a triacylglycerol possesses three polymorphic forms: the amorphous α-form, metastable β′-form, and stable β-form.^22^ These polymorphs differ in that their fatty acid side chain produces different packing in the crystal structure. The α-form possesses a disordered aliphatic chain conformation, which makes it less dense than other forms. It tends to change to a more stable form to reduce the Gibbs free energy of the system. The β′-form has intermediate packing. The transformation from the α-form to the β′-form is faster than the crystallization of the stable β-form without passage through the β′-form. The densest stable β-form, which has the lowest Gibbs free energy, has the highest melting temperature, followed by the β′-form and then the α-form. The DSC thermograms of cetyl palmitate mixed with liquid lipids suggested that the formulations comprised the β′-form and α-form (Figure 3). The DSC thermograms of stearic acid mixed with a liquid lipid showed a broad peak, suggesting the appearance of an amorphous state. The results were confirmed by XRD spectroscopy.

**Table 1:** Melting Onset, Melting Point, Enthalpy Change, and Crystallinity of NLC Formulations from DSC Thermogram at a Solid-to-Liquid Lipid Ratio of 70:30

formulation	melting onset (°C)	melting point (°C)	enthalpy change (J/g)	% CI
cetyl palmitate	48.59	50.93	226.7	100
CC73	43.26	46.41	71.25	45
CI73	45.61	48.04	98.28	62
CO73	44.74	47.52	84.18	53
stearic acid	54.13	57.15	185.2	100
SC73	39.82	47.29	69.01	53
SI73	39.95	48.57	73.02	56
SO73	39.76	47.61	69.17	53

### X-ray Diffraction (XRD) Analysis

**Figure 4:**
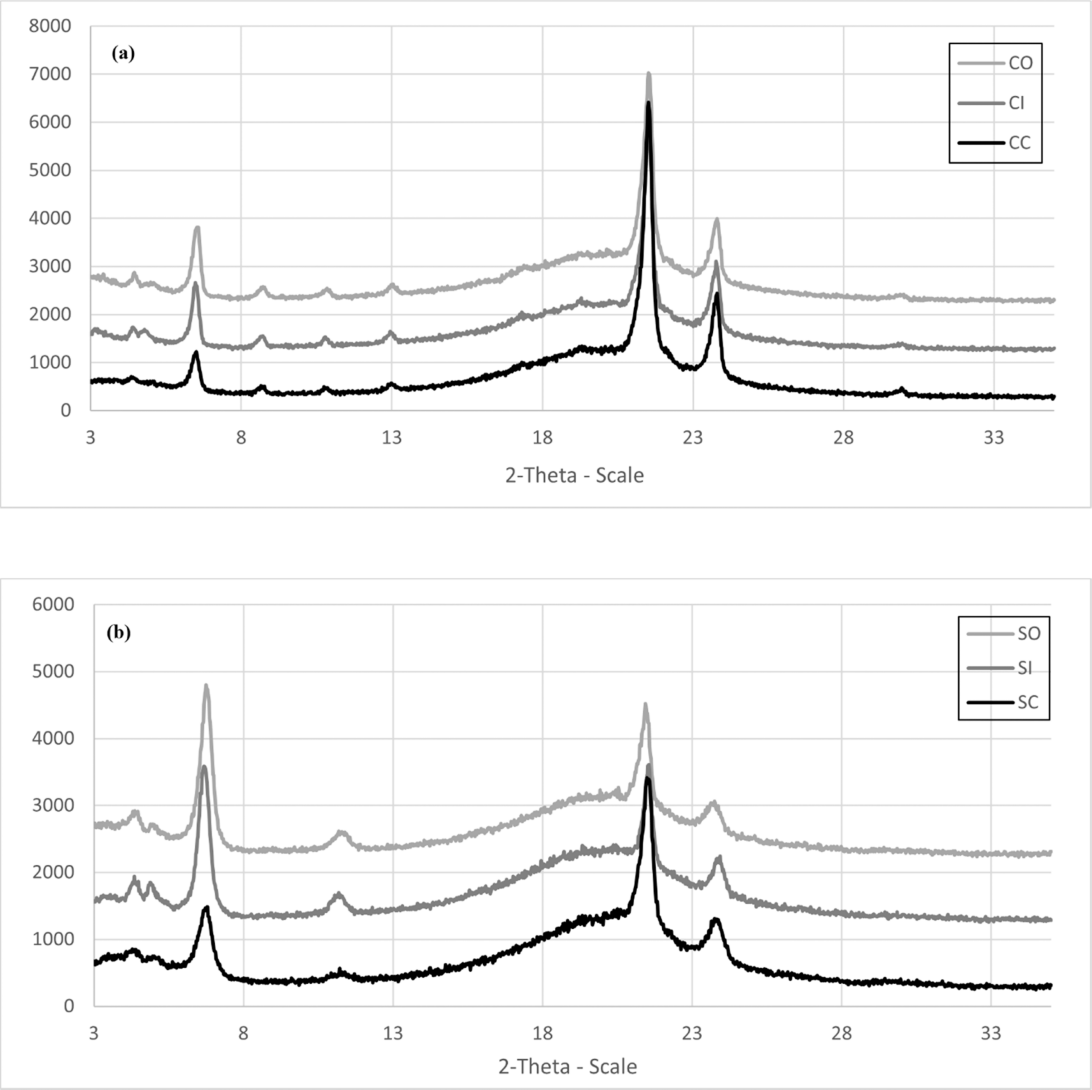
X-ray diffraction (XRD) patterns of NLCs prepared from cetyl palmitate (a) and stearic acid (b) mixed with oleic acid, isopropyl palmitate, and caprylic/capric triglycerides.

X-ray diffraction (XRD) analysis was performed to investigate the polymorphic behavior and crystallinity of the compounds. The diffraction patterns of bulk cetyl palmitate and bulk stearic acid showed shape peaks with no maxima. Bulk cetyl palmitate and bulk stearic acid showed high crystallinity. The diffraction pattern of cetyl palmitate had major peaks at 2θ of 6.5, 21.5, and 23.8° and minor peaks at 2θ of 8.7, 10.8, and 13.0°, while the XRD patterns of stearic acid showed major peaks at the same 2θ of 6.5, 21.5, and 23.8° and a minor peak at 11.2°, indicating orthorhombic lattices (β′ polymorph) with a lattice spacing of 0.38 and 0.42 nm.^22−24^ The XRD patterns of NLC formulations that contained a liquid lipid in the lipid matrix showed a broad curve in all formulations (Figure 4), indicating the appearance of an amorphous form in the lipid matrix. However, peaks at almost the same position also appeared. The NLC formulation should consist of an β′ polymorph mixed with an amorphous form. Solid lipid mixed with caprylic/capric triglycerides had a ratio of peak height at 2θ of 6.5, 21.5, and 23.8°, different from that of solid lipid mixed with isopropyl palmitate or oleic acid.

## Computational Studies

### Analysis of Single Lipid Droplet Systems

**Figure 5:**
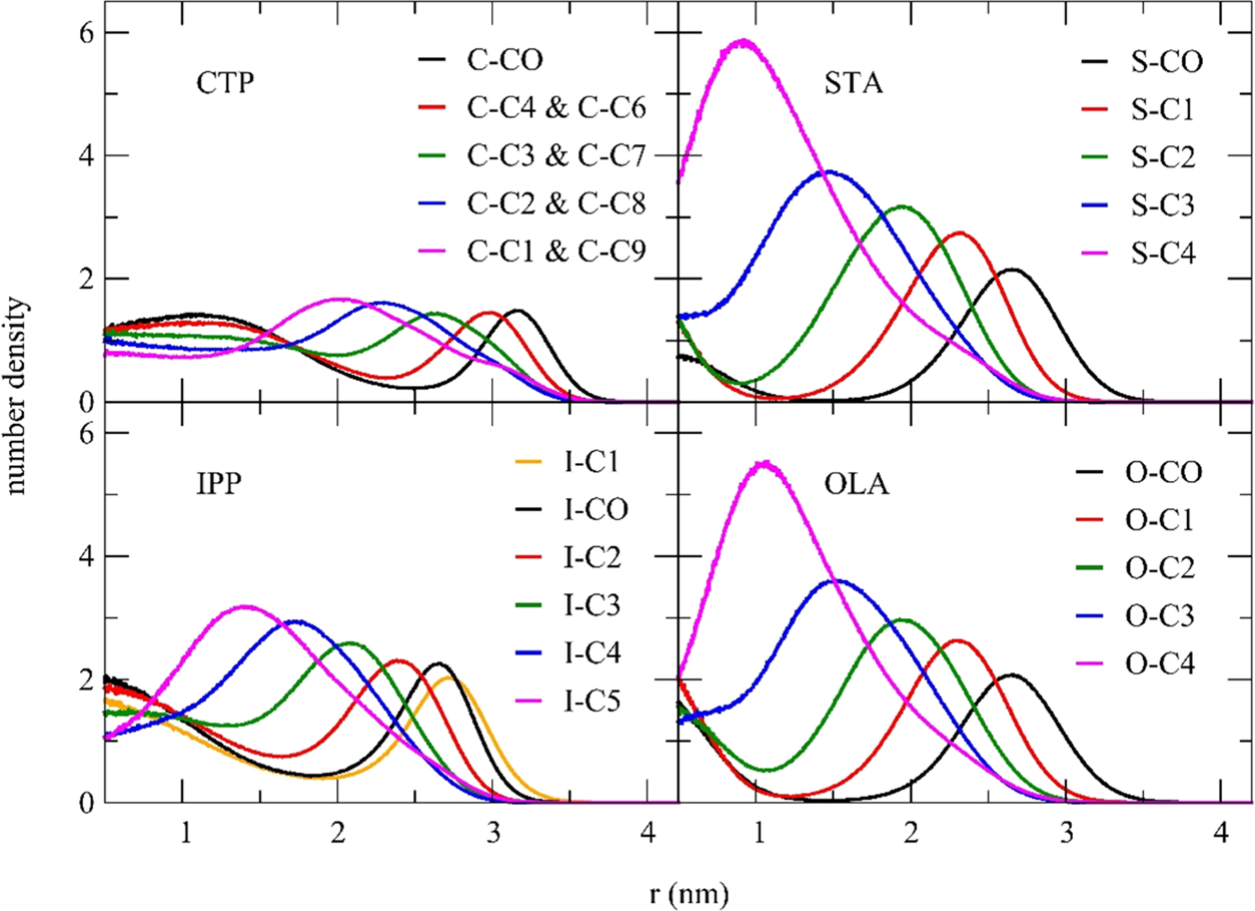
Radial number density for all coarse-grained beads of pure solid lipid: CTP and STA, and pure liquid lipid: IPP and OLA. The CO bead is the most polar in the molecule.

Figure 5 demonstrates the radial number density of single lipid droplet systems. The radial number density was calculated with respect to the geometric center of the droplet. It provides useful information about the preferred locations of the polar and nonpolar parts of the lipid molecules within the droplet. The radial number density shown in Figure 5 indicated that simulated droplets were about 6.5 and 7.0 nm in diameter. The plots for STA and OLA droplets displayed similar features but were different from those for CTP and IPP droplets.

**Figure 6:**
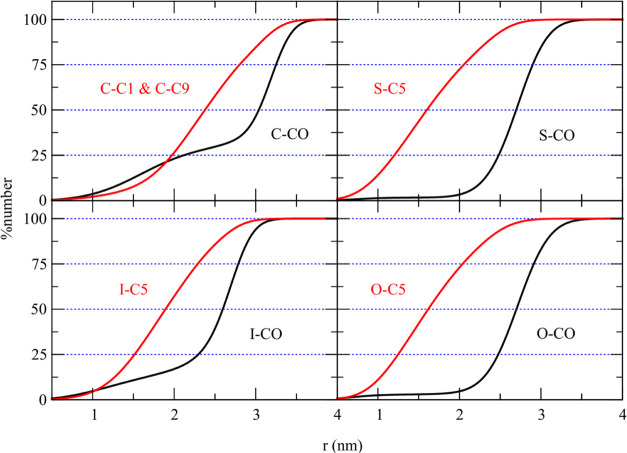
Percentage cumulative number density for the polar (black) and hydrophobic tail (red) beads of pure solid lipid: CTP and STA, and pure liquid lipid: IPP and OLA. The CO bead is the most polar in the molecule.

Both STA and OLA droplets showed similar number density plots due to their highly similar coarse-grained parameters, except for one specific bead containing unsaturated carbons. The radial number density plot showed that the polar group, S–CO and O–CO for STA and OLA droplets, respectively, was essentially localized at the outer region of the droplet. The percentage cumulative number density plots of the polar group (Figure 6) support this observation. They were nearly zero when *r* < 2.3 nm and increased rapidly when *r* > 2.3 nm for both STA and OLA droplets. The radial number density of each nonpolar group shifted its maximum toward the droplet center as the group was farther away from the polar group. The nonpolar group farthest from the polar group, S–C4 or O–C4 for the stearic acid and oleic acid, respectively, were found to be buried inside the droplet, as shown in the radial number density plot. The percentage cumulative number density indicated that approximately 50% of S–C4 and O–C4 were within 1.6 nm of the droplet center. Therefore, the molecules would retain some degree of mobility within the droplet, as this value is much less than 100%.

In contrast to STA and OLA droplets, the CTP and IPP droplets had their polar groups, C–CO and I–CO, respectively, present in both the inner and outer regions of the droplet. The CTP fatty acid ester had the C–CO polar group distributed nonuniformly in both the inner and outer regions of the droplet. As depicted in Figure 6, 25% of the C–CO polar group was buried within the 2 nm radius of the droplet center. At the radial distance of 2–2.8 nm, the percentage cumulative number of the C–CO group increased slowly from 25 to 40%. The remaining 60% of the C–CO group was concentrated at the outermost 0.6 nm of the droplet.

On the other hand, the nonpolar group such as C–C1 and C–C9 were distributed primarily in the middle region of the droplet. For example, the percentage cumulative number density of C–C1 and C–C9 groups rose from 13 to 88% when the radial distance increased from 1.7 to 3 nm. Notably, the cumulative number plot of the nonpolar group reached 100% at a shorter radial distance than that of the polar headgroup, indicating that the CTP droplet’s outermost region in water primarily consisted of the C–CO polar groups.

The IPP liquid fatty acid ester has its I–CO polar group distributed throughout the droplet, similar to the CPP solid fatty acid ester. Figure 6 shows the cumulative number density plot of the I–CO group gradually increasing at small radial distances and then sharply rising at larger distances. Around 75% of the I–CO polar group favored the outer 0.8 nm of the droplet, with the remaining I–CO group buried within the droplet. Most of the I–C5 nonpolar group, the farthest nonpolar group from the I–CO polar group, localized inside the droplet. The cumulative number plot of the I–C5 nonpolar group showed a nearly linear increase with the radial distance from 1.5 to 2.3 nm, reaching 100% at a shorter distance than the I–CO plot. This highlights that the outermost region of the droplet surface primarily consists of the I–CO polar group.

**Figure 7:**
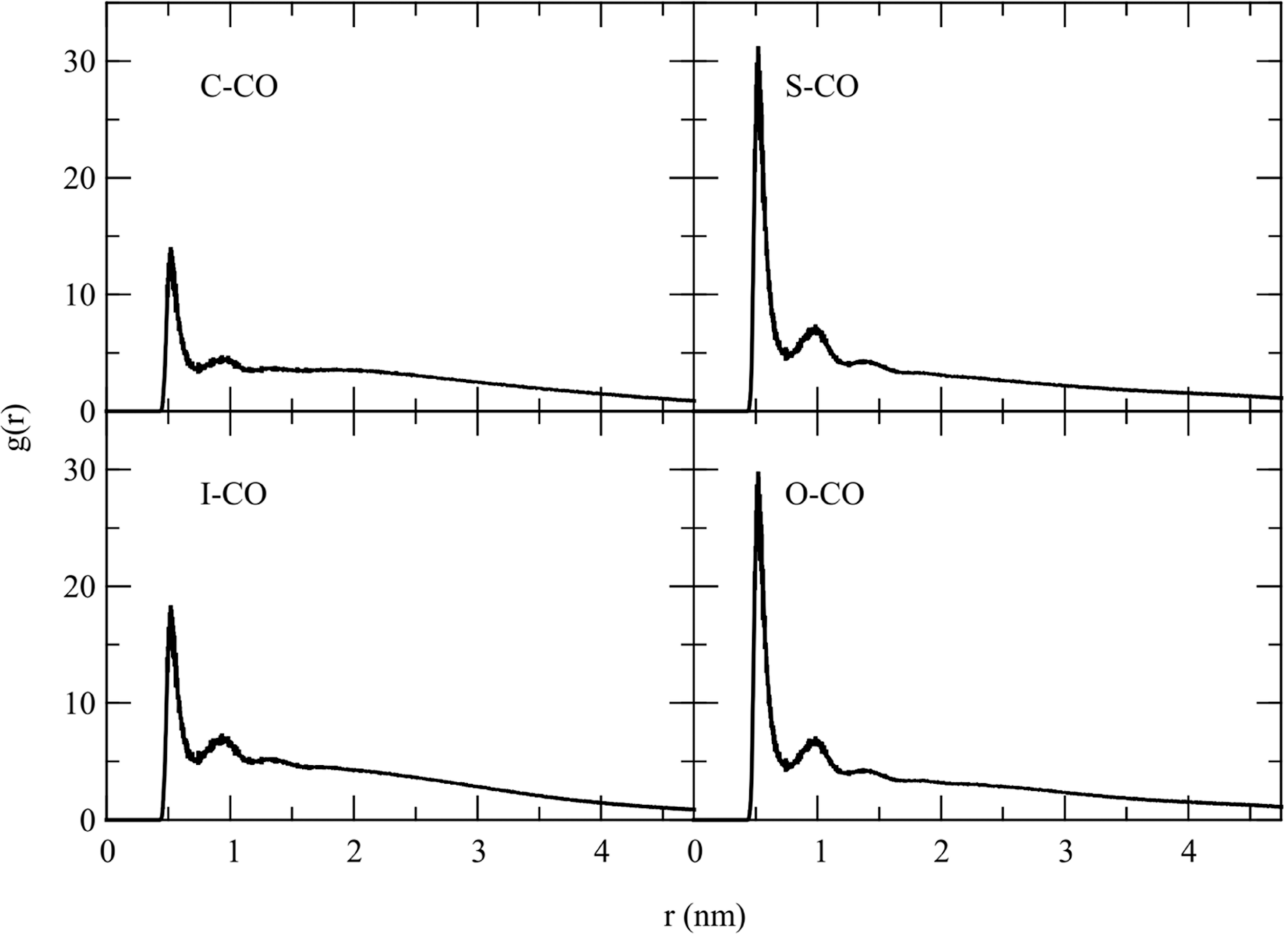
Radial distribution function between coarse-grained polar beads of pure solid lipid: CTP and STA, and pure liquid lipid: IPP and OLA. The CO bead is the most polar in the molecule.

Figure 7 shows the radial distribution function (RDF) of the polar group for all pure lipid droplets in water. The RDF exhibits a sharp peak at around 0.5 nm, corresponding to the closest distance between coarse-grained particles. After this initial peak, the RDF displays a minor secondary peak and then flattens at larger distances. This suggests no long-range ordering within these systems.

### Analysis of Binary Lipid Droplet Systems

**Figure 8:**
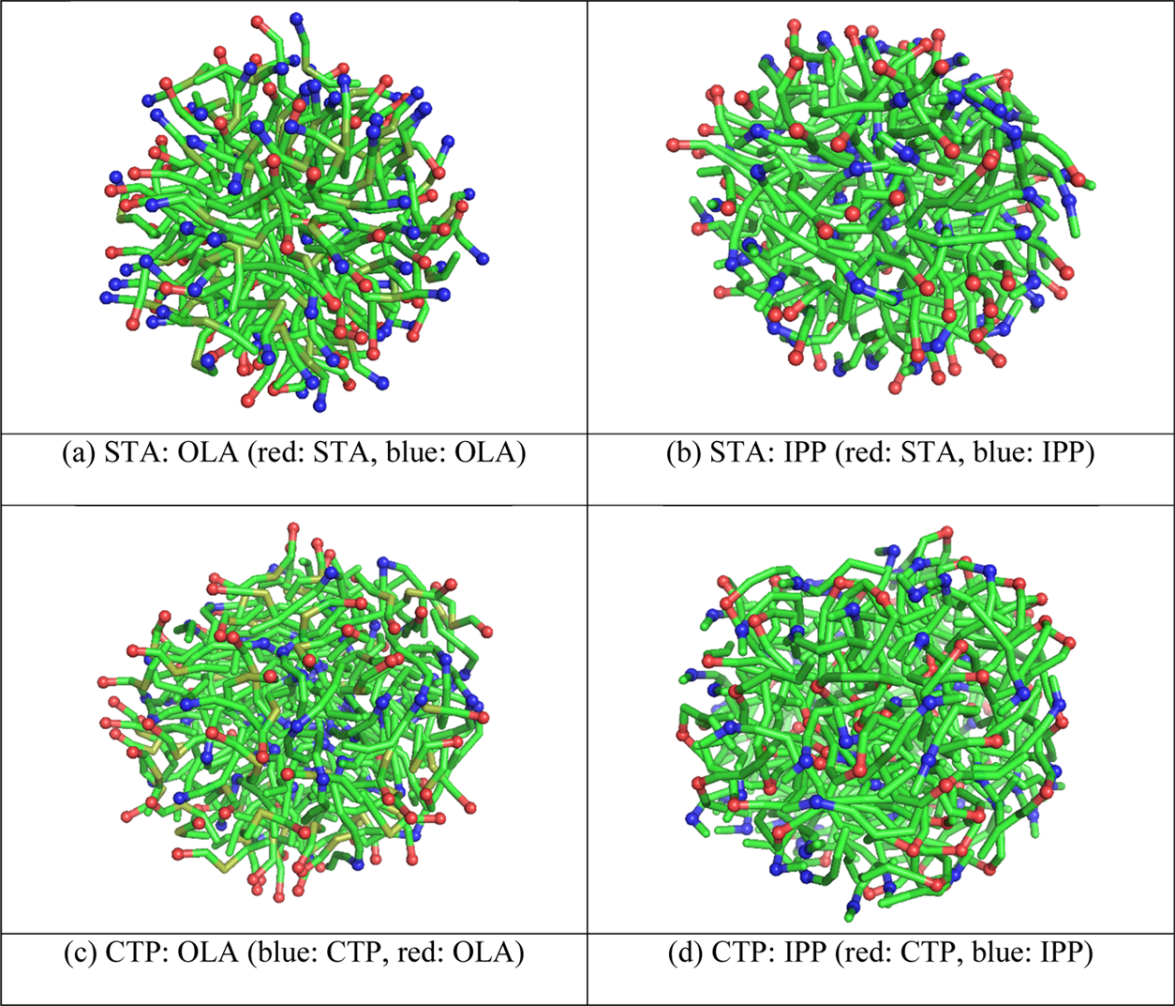
Snapshots of lipid droplet: (a) 50%STA:50%OLA, (b) 50%STA:50%IPP, (c) 50%CTP:50%OLA, and (d) 50%CTP:50%IPP. Red and blue spheres indicate the polar bead of the fatty acid/fatty acid ester.

Figure 8 illustrates the snapshots of binary lipid droplets studied in this work at the 50:50 ratio of solid lipid:liquid lipid. Each lipid molecule was color-coded by its polar bead as described. The snapshots revealed good mixing between lipid molecules of different types. The snapshot also hinted at different distributions of the polar group in each droplet. In an STA:OLA droplet, the polar groups were observed at the outermost region of the droplet. On the other hand, the CTP:OLA droplet exhibited the CTP polar group distributed all over the droplet, while the OLA polar group was concentrated at the outermost region.

**Figure 9:**
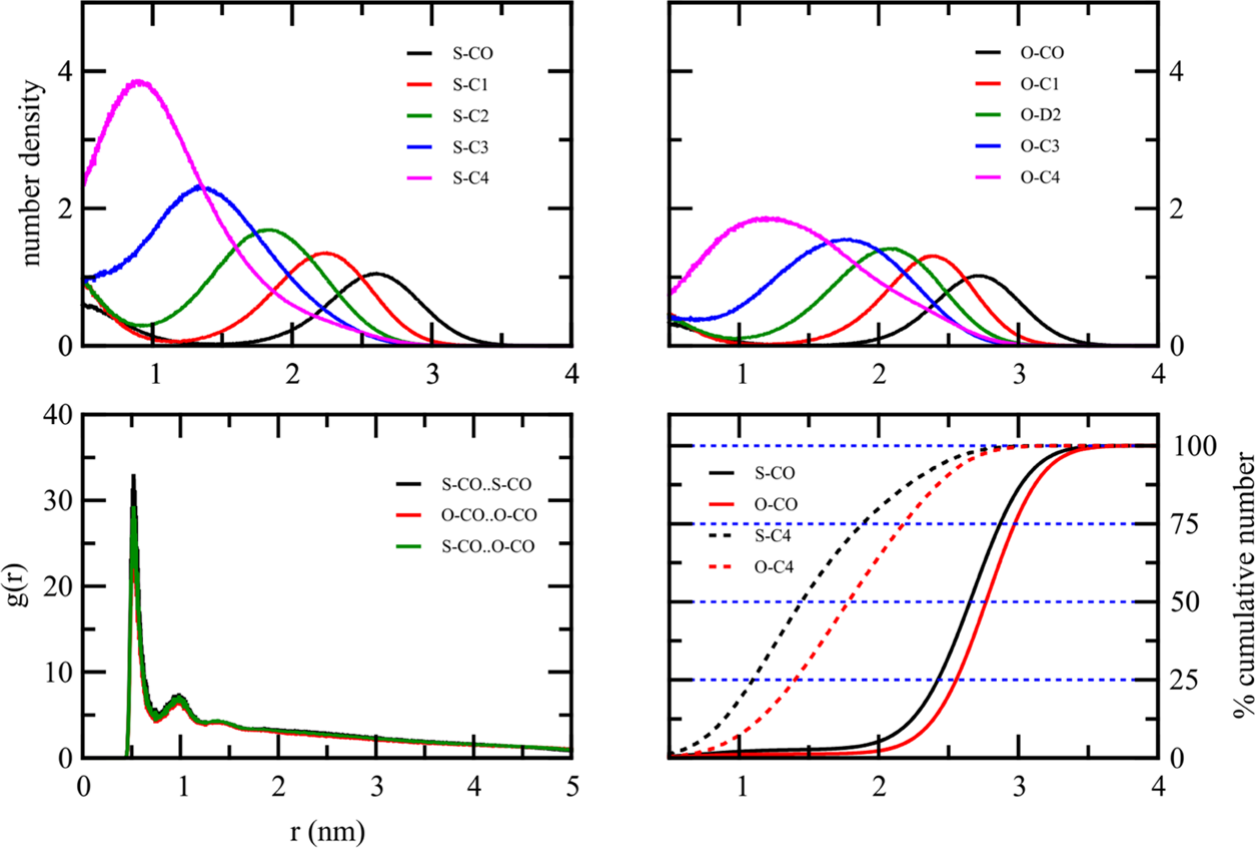
Radial number density, the radial distribution function, and the cumulative radial number density of the 50%STA:50%OLA binary mixture. The CO bead is the polar bead, while the C4 bead is the nonpolar tail bead.

Figure 9 illustrates the radial number density, radial distribution function, and cumulative radial number density of the STA:OLA binary lipid droplet. The RDF of polar groups of the same molecule types, S–CO..S–CO and O–CO..O–CO, were almost identical to that of polar groups of different molecule types, S–CO..O–CO. This suggests a thorough mixing of STA and OLA within the droplet, with the S–CO and O–CO polar groups interchangeable on average. The plot further indicated both STA and OLA polar groups occupy the droplet’s outermost region.

Despite sharing similar structural features with pure droplets, STA and OLA exhibited different spatial arrangements within the binary lipid droplet, as revealed by the radial number density plot. The polar beads of both STA and OLA, as shown by the radial number density, both occupied the outermost region of the droplet. The radial number density of each coarse-grained nonpolar bead in both STA and OLA peaked closer to the center of the droplet as the bead was farther from the polar group. However, the S–C3 and S–C4 plots of STA exhibited sharper peaks compared to those of the O–C3 and O–C4 beads of the OLA. The nonpolar hydrocarbon tail of STA tends to be in the inner region of the droplet compared to that of the OLA. This was confirmed by the cumulative radial number density plot of S–C4 and the O–C4 beads of the STA and the OLA, respectively. The cumulative radial number density plot of S–C4 reached the same value at a shorter distance than that of the atomically compact plot of O–C4. This observation likely stems from the parametrization of the saturated linear hydrocarbon tail of STA in contrast to that of the unsaturated nonlinear tail of OLA mimicking the cis double bond.

**Figure 10:**
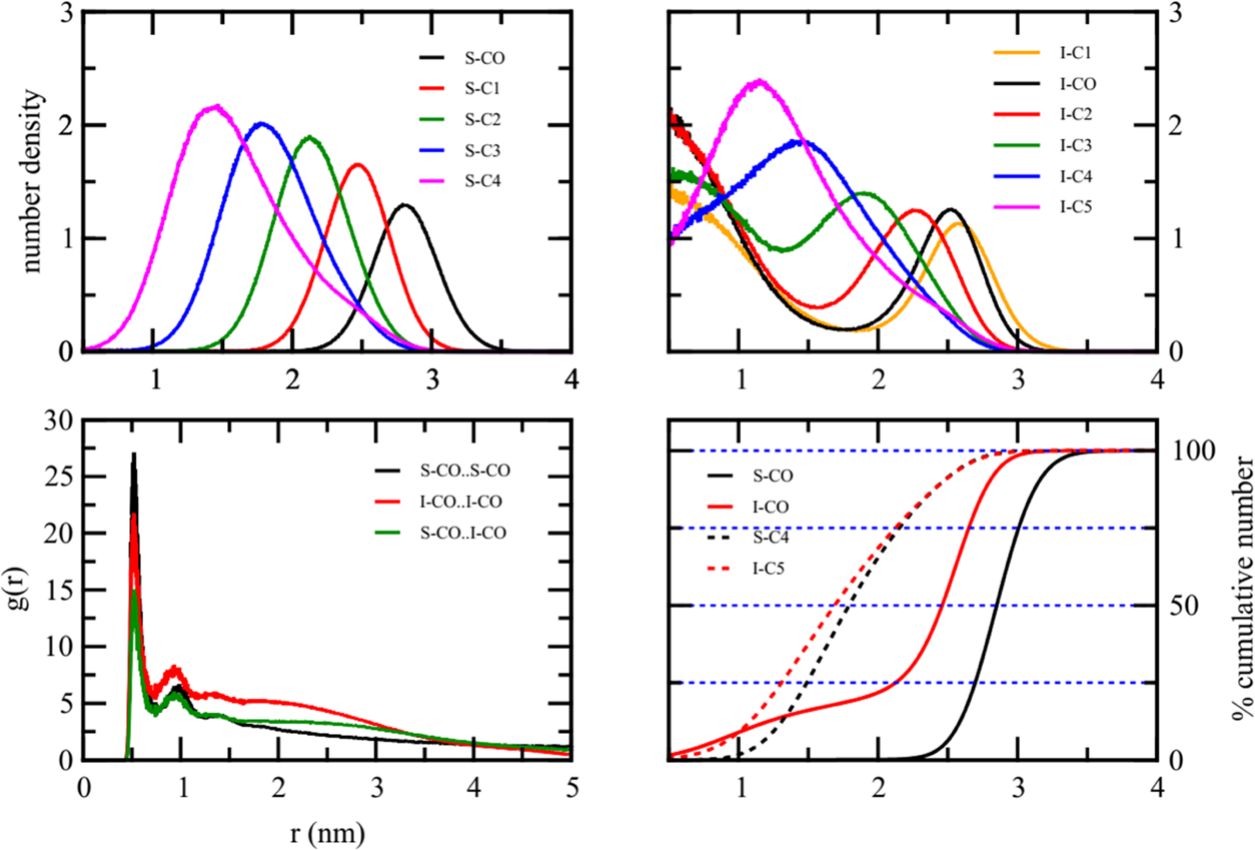
Radial number density, the radial distribution function, and the cumulative radial number density of the 50%STA:50%IPP binary mixture. The S–CO and I–CO beads are the polar beads, while the S–C4 and I–C5 beads are the nonpolar tail beads.

**Figure 11:**
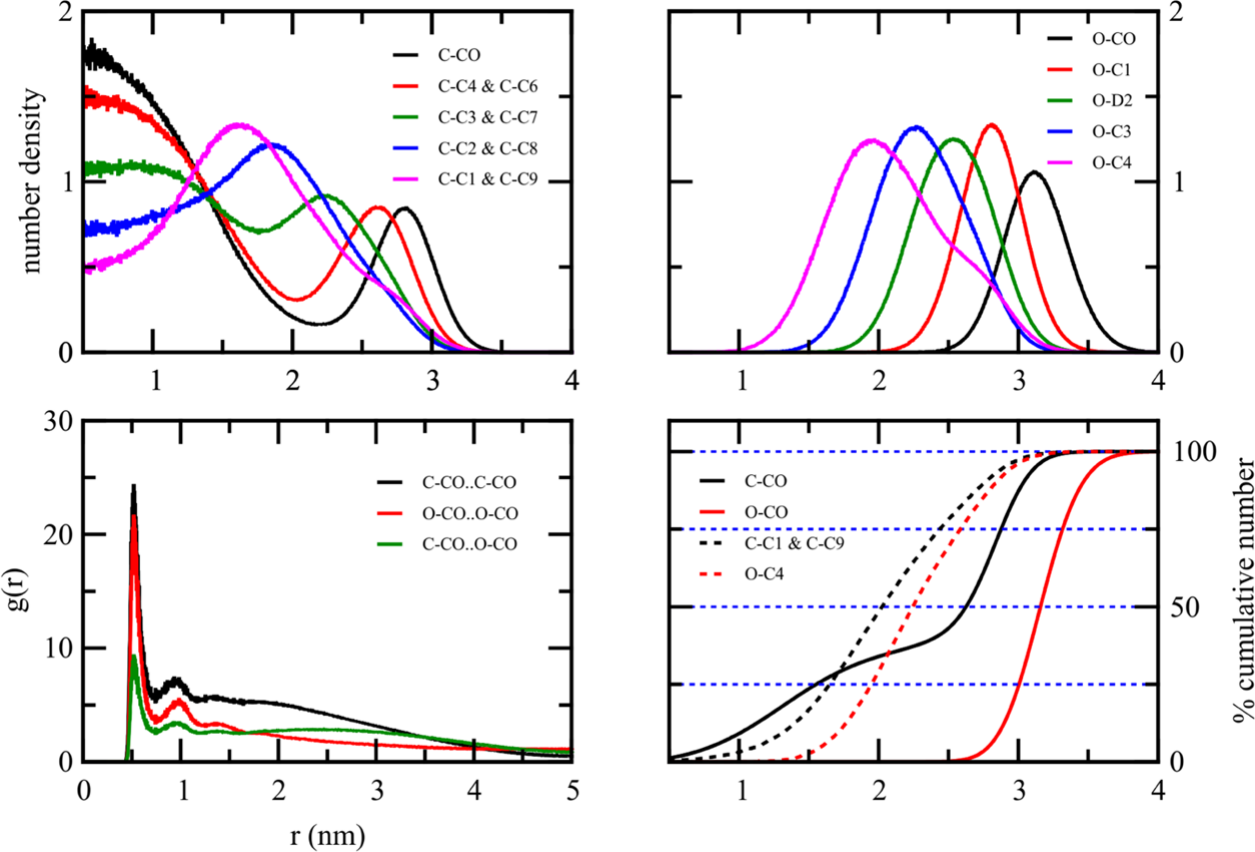
Radial number density, the radial distribution function, and the cumulative radial number density of the 50%CTP:50%OLA binary mixture. The C–CO and O–CO beads are the polar beads, while the C–C9, C–C1, and O–C4 beads are the nonpolar tail beads.

When a binary system comprised a fatty acid lipid and a fatty acid ester, such as STA:IPP or CTP:OLA, the distribution of lipid components in the binary lipid droplet was different from those in the pure lipid droplet. Figures 10 and 11 illustrate important structural parameters of STA:IPP and CTP:OLA at 50%:50%, respectively. For the STA:IPP droplet, the radial number density of STA was more localized than that of the pure STA droplet. At a distance closer to the geometric center, the radial number density of STA falls to zero, while that of IPP is nonzero. As the polar group of STA is more polar than that of IPP, the polar group of STA was closer to the outermost region of the droplet than that of IPP. The radial number density clearly showed that the STA polar group was not found in the innermost region of the droplet. The cumulative number density plot supports this observation as the S–CO reaches 25% within 2.2 nm from the geometric center of the droplet, while the S–CO radial number density plot is flat and close to zero when r is less than 2.3 nm. Beyond this distance, the cumulative S–CO plot then rose sharply to 100%. On the other hand, the IPP radial number density plot indicated that the I–CO polar group was observed at all distances from the droplet center. The I–C1 and I–C2 nonpolar groups, which are adjacent to the I–CO group, had their radial number density like that of I–CO. The I–C1 radial density plot has its maximum almost at the same distance as the I–CO, while the maximum of the I–C2 plot was shifted toward the inner region of the droplet. Therefore, the IPP:STA droplet exhibited the STA polar group at the outermost region, while the IPP molecules were distributed in the inner region of the droplet. Some IPP oriented their I–CO polar group toward the outermost region but cannot penetrate to the surface as compared to the STA polar group. The RDF plot also revealed different arrangements of the STA and IPP polar groups. The RDF of S–CO···S–CO has a prominent first peak at the nearest distance around 0.5 nm. This corresponds to the closest packing. This is followed by a second broad peak around 1 nm. The RDF then slowly dies off. The RDF of I–CO···I–CO has similar features; however, at larger distances, the RDF is greater than that of S–CO..S–CO. The RDF of S–CO···I–CO was intermediate to the other two plots. This implies that at larger distances from one I–CO polar group, the chance of finding another I–CO headgroup is greater than finding another S–CO group. These observations highlight differing distributions of S–CO and I–CO polar groups in the droplet.

For the CTP:OLA droplet, the radial number density of the OLA and CTP (Figure 11) revealed that the OLA positioned the polar group of the O–CO at the outermost region and turned its nonpolar groups to the inner region of the droplet. The radial number density and the cumulative radial number density plots also revealed that the nonpolar groups of OLA never reach the geometric center of the lipid droplet. The innermost region was occupied by CTP molecules. Both the radial number density and the cumulative radial number density plots confirmed that the C–CO polar group of CTP was distributed in all regions of the droplet. The C–CO polar group cannot reach the droplet surface as the C–CO number density peaked at a distance shorter than that of the O–CO polar group of OLA. As in the IPP:STA droplet, the RDF plots of fatty acid and fatty acid ester polar groups were different. The RDF of CTP···CTP polar group was similar to the RDF of IPP···IPP in STA:IPP. At distances greater than 1 nm, the plot was featureless. Its values are greater than the other RDFs shown in the same figure. At larger distances, the plot falls slowly to the value of one. The RDF of OLA···OLA polar group was similar to the RDF of STA in STA:IPP. Drawing parallels with the STA:IPP system, the different behavior of RDF plots confirms the different distributions of CTP and OLA within the droplet.

**Figure 12:**
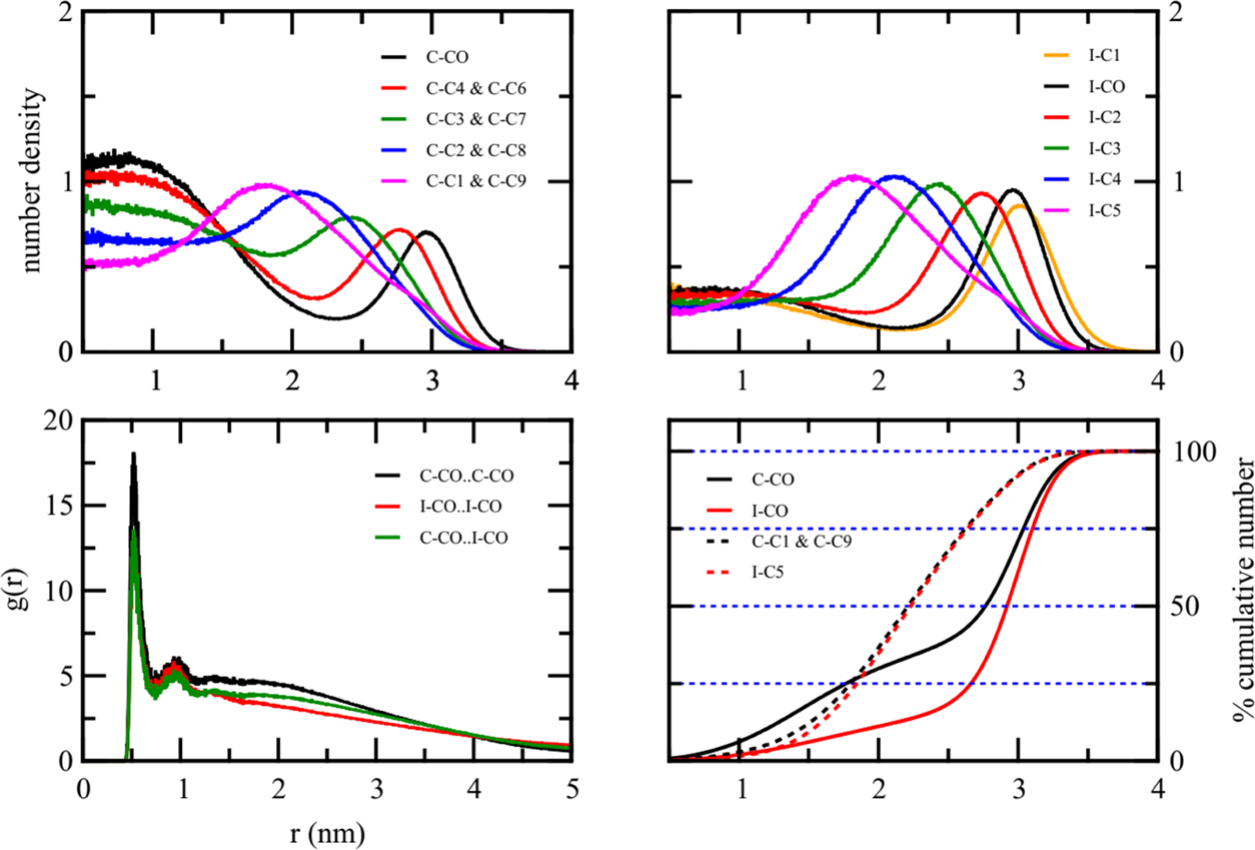
Radial number density, the radial distribution function, and the cumulative radial number density of the 50%CTP:50%IPP binary mixture. The C–CO and I–CO beads are the polar beads, while the C–C9, C–C1, and I–C5 beads are the nonpolar tail beads.

Figure 12 shows some structural parameter plots of the 50%CTP:50%IPP droplet. The radial number density plots of CTP and IPP shared some common features. They indicated that the polar groups, C–CO and I–CO, were observed throughout the droplet. Both C–CO and I–CO polar groups can be observed at the outermost region of the droplet. The I–CO group, however, preferred to be in the outermost region more than the C–CO group. This can be understood from the cumulative radial number density plot. The cumulative plot of I–CO reached 25% at a large distance of 2.7 nm, while that of C–CO was at 1.7 nm. At the outermost region with *r* > 2.7 nm, 75% of the I–CO polar group was observed, while only 55% of the C–CO polar group was found. The radial number density plots of I–CO and C–CO polar groups confirm this observation. The radial number density of the polar group shows two maxims at large and small distances. The radial number density of the C–CO polar group has the maximum at small distances higher than the maximum at large distances. The opposite was observed for the radial number density of the I–CO group. On the other hand, the distribution of nonpolar groups did not have any spatial preference. The cumulative radial number density plots of the nonpolar end group of the two lipid esters are almost identical. The three RDF plots of the polar groups are similar but not identical. All have large RDF values at distances beyond the second peak, with the RDF of C–CO..C–CO having the largest value, followed by those of C–CO..I–CO and I–CO..I–CO. This information indicated that the polar groups were distributed all over the droplet but with different spatial distributions. It also revealed that the C–CO polar groups were found noticeably more inside the droplet than the I–CO groups, which was consistent with the cumulative radial number density plot.

**Figure 13:**
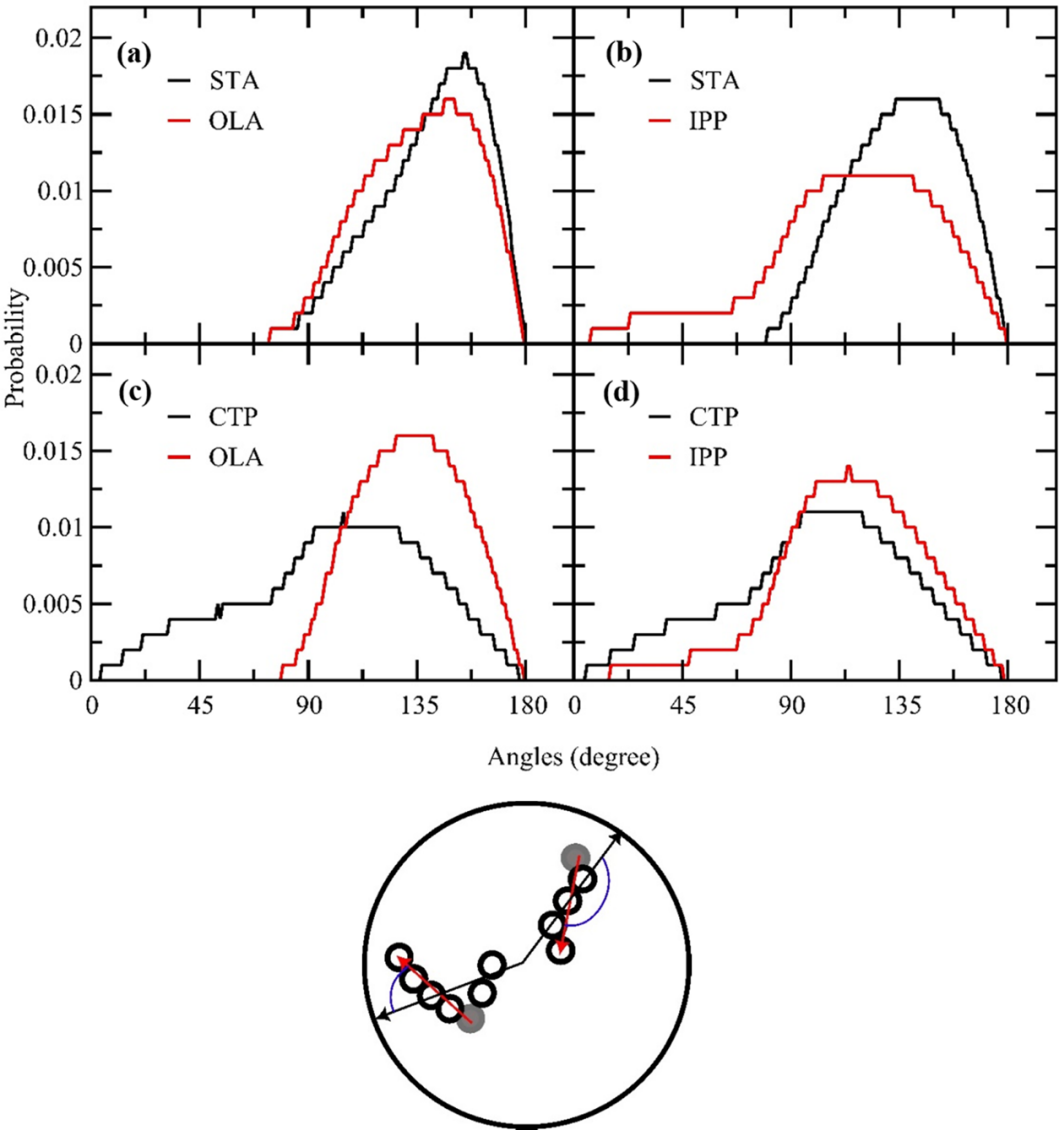
Angular distribution of lipid molecules in lipid droplet: (a) 50%STA:50%OLA, (b) 50%STA:50%IPP, (c) 50%CTP:50%OLA, and (d) 50%CTP:50%IPP. The angle is defined as the angle between the vector extending from the polar bead to the nonpolar tail bead of lipid molecules and the radial vector extending from the geometric center to the midpoint of the first vector.

To understand how the fatty acid and fatty acid ester align themselves within the droplet, we investigated the angular distribution of lipid molecules in the droplet. The angle is defined by the angle between the vector from the polar bead to the nonpolar tail bead of lipid molecules and the radial vector extending from the geometric center to the midpoint of the first vector. Figure 13 illustrates the angular distributions of lipid molecules in all binary lipid droplets. The OLA and STA molecules, which have their polar head mostly on the droplet surface, have an angle distribution peak around 150 and 157°, respectively. The angular distribution is left-skewed, suggesting that all molecules align their polar bead outwardly. The angular distribution of the OLA is slightly broader than that of the STA, which might be due to its double bond parametrization. In contrast, the angular distributions of CTP and IPP differed from those of STA and the OLA. The CTP and IPP molecules oriented their molecular axis in all directions because a broad distribution over all angles was observed. The most probable orientation occurs around the angle of 120–130°, corresponding to where the shallow peak centers. The molecules still preferred to align their polar head in the outward direction, as the angular distribution at angles below 90° is less probable than at angles above 90°. CTP shows an angular distribution similar to that of IPP but with a broader distribution and higher values at angles below 90°. Thus, CTP exhibits greater orientation randomness within the droplet compared to IPP.

## Conclusions

The lipid type, and particularly that of the solid lipid, played a role in the quality of the NLCs, especially the solid lipid. NLCs produced using cetyl palmitate, an ester compound, showed a smaller particle size with greater stability and a lower PDI than those produced using stearic acid, which is a carboxylic acid compound. The liquid lipid type had a smaller influence on the particle size since the solid lipid formed the main structure. The solid/liquid lipid ratio affected the particle size when stearic acid was used, suggesting that the lipid type determines the quality of the NLCs. Simulations showed a link between the phase behavior of the lipid mix and the quality of the prepared NLCs. In future research, the interaction between lipid droplets and the surfactant should be simulated to mimic a system that is closer to the real system currently used for NLC preparation.

## Materials and Methods

### Materials

Cetyl palmitate, isopropyl palmitate, and caprylic/capric triglycerides were purchased from the Namsiang Group (Bangkok, Thailand). Oleic acid, stearic acid, Tween80 (Polysorbate 80), and Span40 (Sorbitan monopalmitate) were purchased from Sigma-Aldrich (Japan). Distilled water and deionized water were prepared freshly using an Autostil 8000× (England) and aquaMax-Ultra (Korea), respectively. All other chemicals used were commercial products of analytical grade. The materials were used without further purification.

### Preparation of Nanostructured Lipid Carriers (NLCs)

Nanostructured lipid carriers (NLCs) were prepared by melt-emulsification using a high-speed homogenizer (Homogenizer SC200010341, Germany). The lipid phase and aqueous phase were prepared separately by heating to 80 °C. The aqueous phase was then added to the lipid phase and homogenized at 14,000 rpm for 3 min. The prepared formulations were stored at 4 °C and room temperature for further study. The lipid phase contained a solid lipid, a liquid lipid, and Span40 as a lipophilic surfactant, while the aqueous phase contained Tween80 as a hydrophilic surfactant in water. The NLCs were prepared using two solid lipid types, cetyl palmitate and stearic acid, and three liquid types, oleic acid, isopropyl palmitate, and caprylic/capric triglycerides, at solid/liquid ratios of 50:50, 70:30, and 90:10, respectively. A mixture of Span40 (a lipophilic surfactant) and Tween80 (a hydrophilic surfactant) at a ratio of 1:1 was used as a cosurfactant.

### Particle Size Measurement by Dynamic Light Scattering (DLS)

Particle size analysis was performed by using dynamic light scattering (DLS) (Malvern Zetasizer Nano-ZS, Malvern Instruments, U.K.). The samples were prepared by 10 times dilution with deionized (DI) water and measured at a scattering angle of 173° in a folded capillary cell (DTS1060). The mean particle size, polydispersity index (PI), and ζ-potential were obtained by the average of five measurements. All measurements were made in triplicate.

### Differential Scanning Calorimetry (DSC)

The degree of crystallinity of the NLCs was derived using differential scanning calorimetry (DSC Q200, TA Instruments). Before measurement, the prepared NLCs were lyophilized for 24 h to eliminate water from the samples. Each sample was then placed in an aluminum pan with an empty pan as a reference. The samples were scanned from 0 to 85 °C at a heating rate of 5 °C/min and then cooled to 0 °C at the same rate over two cycles. The melting onset temperature, melting point temperature, and melting enthalpy were determined. The crystallinity index (%CI) was calculated using eq 1.
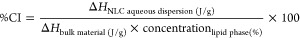
1

### X-ray Diffraction (XRD) Analysis

After the NLC suspensions were lyophilized, X-ray diffraction (XRD) analysis was performed (AXS Model D8 Discover; Bruker, Germany) at room temperature. A copper anode (Cu Kα radiation) was used as the X-ray source. The sample was scanned from 3 to 35° with a scan speed of 0.2 s/step at 40 mA and 40 kV.

### Coarse-Grained Molecular Dynamics Simulation (CG-MD)

To understand the lipid droplet at the molecular level, CG-MD simulations were carried out for lipid droplets with single and binary components. The binary mixture was prepared by mixing either fatty acid or fatty ester, which was a waxy solid at room temperature, with those that were oily liquids at room temperature. The neutral fatty acids used in this study were stearic acid (STA) and oleic acid (OLA). The neutral fatty acid esters consisted of cetyl palmitate (CTP) and isopropyl palmitate (IPP). At room temperature, both stearic acid and cetyl palmitate are waxlike solids, while the others are oily liquids.

**Figure 14:**
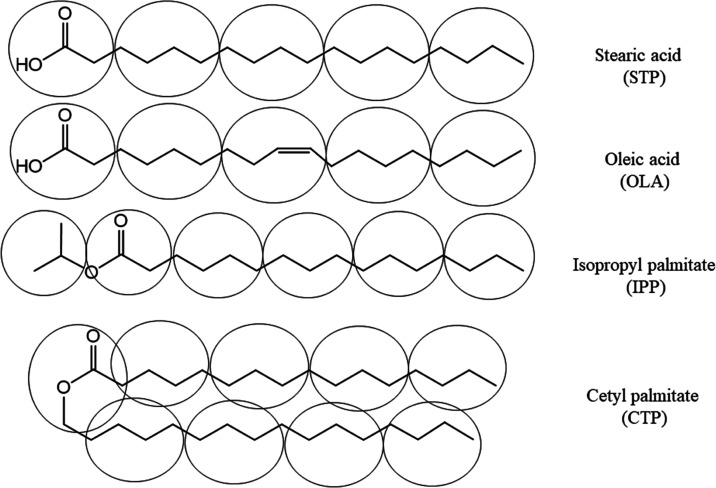
Coarse-grain model of fatty acids and fatty esters considered in this work.

In addition to single lipid systems, four lipid mixtures were prepared: STA:OLA, STA:IPP, CTP:OLA, and CTP:IPP. For each binary mixture, several ratios of waxy solid: oily liquid with ratios ranging from 10:90 to 90%:10% were considered. Since no significant changes were observed during simulations, we report only the 50%:50% systems in this work. Typical systems comprised 150 fatty acid/fatty acid ester molecules in a 10 × 10 × 10 nm^3^ cubic box of water. The coarse-grained MARTINI force fields were adopted to speed up the calculations.^14−16^Figure 14 shows the coarse-grained model of fatty acids and fatty esters considered in this work. As suggested by Janke et al.,^17^ the MARTINI parameter of fatty acid was coupled with the carboxylic acid parameter from the aspartate side chain for the free fatty acid parametrization.^17^ The coarse-grained MARTINI force field W2.0 was used to represent a cluster of waters.^25^ A typical system contained about 8400–9000 coarse-grained water molecules.

Initially, all molecules were placed randomly in the simulation box. Energy minimization was carried out on the initial random configuration. Then, molecular dynamics simulation was carried out while slowly increasing the system temperature until the temperature reached room temperature of 298 K. Afterward, the NPT simulation was performed on the system for 3.5 ms (μs) until it reached equilibrium. The velocity Verlet algorithm was used with a time step of 20 fs. The v-rescale thermostat with a coupling constant of 0.1 ps was employed to keep the temperature constant at 298 K.^26^ Isotropic pressure coupling with a reference pressure of 1.0 bar was maintained using the Berendsen coupling method with a coupling constant of 0.5 ps and a compressibility of 6 × 10^–5^ bar^–126^. The sampling period during the production run is at least 4 μs.

To analyze the fatty acid/fatty acid ester arrangement in the nanodroplet, the droplet was recentered into the original simulation box. Using the gromacs rdf function, the radial number density from the geometric center of the droplet was analyzed. The radial number density plots were evaluated for each type of coarse-grained bead. In MARTINI terminology, the polar group is represented as a CO. The radial distribution functions between the polar groups of the same and different molecule types were also computed. The angular distribution of lipid molecules in lipid droplets was used to describe lipid molecule orientation relative to the radial vector. The angle is defined by the angle between the vector from the polar bead to the nonpolar tail bead of lipid molecules and the radial vector extending from the geometric center to the midpoint of the first vector.

All simulations were carried out using GROMACS 5.1.5 and 2016.6.^27^ PyMOL was used for the visualization of the droplet.

### Statistical Analysis

The particle size measurements were presented as the mean ± the standard deviation (SD). The statistical significance of differences was examined using a one-way ANOVA at a probability level of 0.05. The Tukey HSD (Honestly Significant Difference) posthoc test was applied to identify significant differences between groups at a 95% confidence interval.
